# Supplementary material for: A pH-sensitive motif in an outer membrane protein activates bacterial membrane vesicle production
Source: Nat Commun. 2024 Aug 13;15:6958. doi: 10.1038/s41467-024-51364-z (PMC11322160; doi:10.1038/s41467-024-51364-z)
Supplement: Supplementary file 1 — Supplementary information [file 41467_2024_51364_MOESM1_ESM.pdf]

A pH-sensitive motif in an outer membrane protein activates bacterial membrane vesicle production

Ruchika Dehinwal[a], Tata Gopinath[b], Richard D. Smith[c], Robert K Ernst[c], Dieter M. Schifferli[d,#], Matthew K. Waldor[a,#], Francesca M. Marassi[b,#]

[a] Division of Infectious Diseases, Brigham and Women's Hospital, United States; Department of Microbiology, Harvard Medical School, United States; Howard Hughes Medical Institute, United States.

[b] Department of Biophysics, Medical College of Wisconsin 8701 Watertown Plank Road, Milwaukee, WI 53226-3548 USA

[c] Department of Microbial Pathogenesis, School of Dentistry, University of Maryland, Baltimore, Maryland 21201, USA

[d] Department of Pathobiology, University of Pennsylvania, School of Veterinary Medicine, Philadelphia, Pennsylvania, USA

Table S1. Strains, plasmids and primers used in this study

Strain	Plasmid	Description	Antibiotics	Reference
WT	-	SL1344 fliC::Cmr , fljB::MudJ	Str^r, Km^r, Cm^r	(1)
Δ*pagC*	-	*pagC* deletion in WT	Str^r, Km^r, Cm^r	(1)
PagC(R48A)	-	PagC R48 replaced by alanine	Str^r, Km^r, Cm^r	This work
PagC(H60A, H62A, K64A, K68A)	-	PagC H60, H62, K64 and K68 replaced by alanine	Str^r, Km^r, Cm^r	This work
PagC(K96A, K100A, H102A)	-	PagC K96, K100 and H102 replaced by alanine	Str^r, Km^r, Cm^r	This work
PagC(K113A, R117A, K118A)	-	PagC K113, R117 and K118 replaced by alanine	Str^r, Km^r, Cm^r	This work
PagC(R48A, K113A, R117A, K118A)	-	PagC R48, K113, R117 and K118 replaced by alanine	Str^r, Km^r, Cm^r	This work
PagC(R48A, K96A, K100A, H102A)	-	PagC R48, K96, K100 and H102 replaced by alanine	Str^r, Km^r, Cm^r	This work
PagC(K96A, K100A, H102A, K113A, R117A, K118A)	-	PagC K96, K100, H102, K113, R117, and K118 replaced by alanine	Str^r, Km^r, Cm^r	This work
ΔpagCΔrckΔompXΔpgtE	pRS1	*ΔpagCΔrckΔompXΔpgtE* mutant bacteria with empty vector	Str^r, Km^r, Tet^r, Amp^r	(1)
ΔpagCΔrckΔompXΔpgtE	p*pagC*	*ΔpagCΔrckΔompXΔpgtE* mutant expressing wildtype PagC protein	Str^r, Km^r, Tet^r, Amp^r	(1)
ΔpagCΔrckΔompXΔpgtE	p*rck*	*ΔpagCΔrckΔompXΔpgtE* mutant expressing wildtype Rck protein	Str^r, Km^r, Tet^r, Amp^r	(1)
ΔpagCΔrckΔompXΔpgtE	p*pagC-rck*$^{2\text{-}3}$	*ΔpagCΔrckΔompXΔpgtE* mutant expressing chimeric PagC protein with EL2'-3' of Rck	Str^r, Km^r, Tet^r, Amp^r	This work
ΔpagCΔrckΔompXΔpgtE	p*rck-pagC*$^{2\text{-}3}$	*ΔpagCΔrckΔompXΔpgtE* mutant expressing chimeric Rck protein with EL2-3 of PagC	Str^r, Km^r, Tet^r, Amp^r	This work
ΔpagCΔrckΔompXΔpgtE	p*rck-pagC*^{3}	*ΔpagCΔrckΔompXΔpgtE* mutant expressing chimeric Rck protein with EL3 of PagC	Str^r, Km^r, Tet^r, Amp^r	This work
ΔpagCΔrckΔompXΔpgtE	p*rck-pagC*^{2}	*ΔpagCΔrckΔompXΔpgtE* mutant expressing chimeric Rck protein with EL2 of PagC	Str^r, Km^r, Tet^r, Amp^r	This work
ΔpagCΔrckΔompXΔpgtE	p*rck-pagC*^{2}_H60A	*ΔpagCΔrckΔompXΔpgtE* mutant expressing chimeric Rck protein with EL2 of PagC and His60 substituted by Ala	Str^r, Km^r, Tet^r, Amp^r	This work
ΔpagCΔrckΔompXΔpgtE	p*rck-pagC*^{2}_H62A	*ΔpagCΔrckΔompXΔpgtE* mutant expressing chimeric Rck protein with EL2 of PagC and His62 substituted by Ala	Str^r, Km^r, Tet^r, Amp^r	This work
ΔpagCΔrckΔompXΔpgtE	p*rck-pagC*^{2}_H60A + H62A	*ΔpagCΔrckΔompXΔpgtE* mutant expressing chimeric Rck protein with EL2 of PagC and His60 and 62 substituted by Ala	Str^r, Km^r, Tet^r, Amp^r	This work

ΔpagCΔrckΔompXΔpgtE	p*pagC*_H60K	*ΔpagCΔrckΔompXΔpgtE* mutant expressing PagC protein with and His60 substituted by Lys	Str^r, Km^r, Tet^r, Amp^r	This work
ΔpagCΔrckΔompXΔpgtE	p*pagC*_H62K	*ΔpagCΔrckΔompXΔpgtE* mutant expressing PagC protein with and His62 substituted by Lys	Str^r, Km^r, Tet^r, Amp^r	This work
ΔpagCΔrckΔompXΔpgtE	p*pagC*_H60K +H62K	*ΔpagCΔrckΔompXΔpgtE* mutant expressing PagC protein with and His60 and 62 substituted by Lys	Str^r, Km^r, Tet^r, Amp^r	This work
ΔpagCΔrckΔompXΔpgtE	p*pagC*_H60K +H62K +H102K	*ΔpagCΔrckΔompXΔpgtE* mutant expressing PagC protein with and His60, 62 and 102 substituted by Lys	Str^r, Km^r, Tet^r, Amp^r	This work

**Primers**	**Sequence (5' to 3')**	**Description**
PagC.R48.R	GCCTGTGCGTCTCCATATA	For cloning *pagC* R48A
PagC.R48.F	TATATGGAGACGCACAGGC	"
PagC.H60,H62,K64,K68.R	GCCACCTCAAACGCGTCAGCGTAAGC	For cloning *pagC* H60A H62A K64A K68A
PagC.H60,H62,K64,K68.F	GCTTACGCTGACGCGTTTGAGGTGGC	"
PagC.K96,K100,H102.R	GGAAGCTTCTGCAAATGTCGCCGCTAC	For cloning *pagC* K96A K100A H102A
PagC.K96,K100,H102.F	GTAGCGGCGACATTTGCAGAAGCTTCC	"
PagC.K113,R117,K118.R	TAACGCAATTTCCTCAGCGGCAACGG	For cloning *pagC* K113A R117A K118A
PagC.K113,R117,K118.F	GCAATTTCCTCAGCGGCA	"
mutantPagC.F	CCGTAAATAATAAGTAGTATTAAGGAGTTGTTATGAAA AATATTATTTTATCCACTTTAG	For amplifying *pagC*, with overhangs for *pagC* upstream region
mutantPagC.R	GCTTTTCAGAAACGGTATCCAACCCCGACG	For amplifying *pagC*, with overhangs for *pagC* downstream region
pOPC3-900.F	ACGGTATCGATAAGCTTGATATCGAATTCTGTTGTAA ACCAGAACAATGGC	For amplifying 900bp region upstream of *pagC*, with plasmid overhangs
pOPC3-900.R	CTAAAGTGGATAAAATAATATTTTTCATAACAACTCCT TAATACTACTTATTATTTACGG	For amplifying 900bp region upstream of *pagC*, with overhangs for *pagC*
pOPC3+900.F	CAACGTCGGGGTTGGATACCGTTTCTGAAAAGCATAA GCTATG	For amplifying 900bp region downstream of *pagC*, with overhangs for *pagC*
pOPC3+900.R	GGCCGCTCTAGAACTAGTGGATCCTCTGCTATTGATC GTATC	For amplifying 900bp region downstream of *pagC*, with plasmid overhangs
PagC EL2.F	GCGTCGTTCAGTTGGTTATATGGAGACAGACAGGCT	For amplifying *pagC* EL2
PagC EL2.R	CCGCCATCAGCGAACCGTACTTCACCTCAAAC	"
PagC EL3.F	CGGTGCCGGTACCGTAAAGGCGACATTTAAAGAA	For amplifying *pagC* EL3
PagC EL3.R	TTCTTTAAATGTCGCCTTTACGGTACCGGCACCG	"

PagC EL2-3.F	ATTCTCCAGCGGATTC	For amplifying EL2 to EL3 of *pagC*
PagC EL2-3.R	CTCATAGCCCAGATCGATGACCACATTCTCCAGCGG ATTCATCTG	“
PagC_5’.F	AGCGGTGAATTATTCGTGGTATGAAAAATATTATTTTA TCCACTTTAG	for amplifying *pagC* from the start codon, with pRS1 plasmid overhangs
PagC_5’.R	GCCATCAGTCCCAGCGGCGTCTGAGCCTCATAACGG TATTTCACATTTA	for amplifying *pagC* until EL2 start region
PagC_3’.F	GGCGTACAGTTTAATCCGGTGGAAAATATCGTCGTCG ATGTTGGGTATGAAGG	for amplifying *pagC* from EL3 end region
PagC_3’.R	ATCCGCCAAAACAGCCATCAGAAACGGTATCCAACC CCGACG	For amplifying *pagC* 3’ end, with pRS1 plasmid overhangs
Rck EL2-3.F	TAAATGTGAAATACCGTTATGAGGCTCAGACGCCGCT GGGACTGATGGC	For amplifying EL2 to EL3 of *rck*
Rck EL2-3.R	CCTTCATACCCAACATCGACGACGATATTTTCCACCG GATTAAACTGTACGCC	“
Rck_5’.F	CACTCCCTATCAGTGATAGAGAAAAGTGCATGAAAAA AATCGTTCTGTCCTCA	For amplifying 5’ region of *rck*, with pRS1 plasmid overhangs
Rck_5’.R	AATAAAACTTACCGGAGAGTCATCCTCATAGCGGTAT TTCAGGTTC	For amplifying *rck* until EL2 start region
Rck_3’.F	TGTACAGATGAATCCGCTGGAGAATGTGGTCATCGAT CTGG	For amplifying *rck* from the EL3 3’ end
Rck_3’.R	ATCCGCCAAAACAGCCATCAGAACCGGTAACCG	For amplifying 3’ region of *rck*, with pRS1 plasmid overhangs
Rck_3’long.R	GTTTGAGGTGAAGTACGGTTCGCTGATGGCGG	For amplifying *rck* from the EL2 end region
Rck_5’long.R	GGCACCGGCCAGTGCATACAGAGATA	For amplifying *rck* until EL3 start region
Rck_5’.R	AGCCTGTCTGTCTCCATATAACCAACTGAACGACGC	For amplifying 5’ region of *rck* until EL2 start region
H60A.F	GTCTGTTGAGCCTGAAGGTATTGCTTACCATGACAAG TTTGAGGTGAAGTAC	For substituting PagC EL2 His-60 by Ala
H60A.R	GTACTTCACCTCAAACTTGTCATGGTAAGCAATACCT TCAGGCTCAACAGAC	“
H62A.F	GTCTGTTGAGCCTGAAGGTATTCATTACGCTGACAAG TTTGAGGTGAAGTAC	For substituting PagC EL2 His-62 by Ala
H62A.R	GTACTTCACCTCAAACTTGTCAGCGTAATGAATACCT TCAGGCTCAACAGAC	“
H60A,H62A.F	GTCTGTTGAGCCTGAAGGTATTGCTTACGCTGACAAG TTTGAGGTGAAGTAC	For substituting PagC EL2 His-60 and 62 by Ala
H60A,H62A.R	GTACTTCACCTCAAACTTGTCAGCGTAAGCAATACCT TCAGGCTCAACAGAC	“
H60K.F	GGTCTGTTGAGCCTGAAGGTATTAAATACCATGACAA GTTTGAGGTGAAGTACGG	For substituting PagC EL2 His-60 by Lys
H60K.R	CCGTACTTCACCTCAAACTTGTCATGGTATTTAATACC TTCAGGCTCAACAGACC	“
H62K.F	GGTCTGTTGAGCCTGAAGGTATTCATTACAAAGACAA GTTTGAGGTGAAGTACGG	For substituting PagC EL2 His-62 by Lys

H62K.R	CCGTACTTCACCTCAAACTTGTCTTTGTAATGAATACC TTCAGGCTCAACAGACC	“
H60,H62K.F	GGTCTGTTGAGCCTGAAGGTATTAAATACAAAGACAA GTTTGAGGTGAAGTACGG	For substituting PagC EL2 His-60 and 62 by Lys
H60,H62K.R	CCGTACTTCACCTCAAACTTGTCTTTGTATTTAATACC TTCAGGCTCAACAGACC	“

Table S2. Systems for MD simulation. All simulations were initiated form AlphaFold: model P23988, with either neutral or protonated His residues.

System	OM Top Leaflet	OM Bottom Leaflet	System size	Number of Ions	Number of water molecules
**1** neutral His	STm LPS: 25	DMPC:57 DMPG:20	74 x 74 x 115	Cl^-:22 K^+: 43 Ca^{2+}:125	10,745
**2** neutral His	STm LPS: 25	DMPC:57 DMPG:20	74 x 74 x 119	Cl^-:23 K^+: 44 Ca^{2+}:125	11,176
**3** neutral His	STm LPS: 25	DMPC:57 DMPG:20	73 x 73 x 123	Cl^-:19 K^+: 40 Ca^{2+}:125	11,461
**4** protonated His	STm LPS: 25	DMPC:57 DMPG: 20	74 x 74 x 121	Cl^-:23 K^+: 41 Ca^{2+}:125	11,796
**5** protonated His	STm LPS: 25	DMPC:57 DMPG:20	74 x 74 x 120	Cl^-:23 K^+: 41 Ca^{2+}:125	11,262
**6** protonated His	STm LPS: 25	DMPC:57 DMPG:20	73 x 73 x 123	Cl^-:23 K^+: 41 Ca^{2+}:125	11,683
**7** neutral His	DMPC:57 DMPG: 20	DMPC:57 DMPG:20	74 x 74 x 122	Cl^-:44 K^+: 85	13,597
**8** neutral His	DMPC:57 DMPG: 20	DMPC:57 DMPG:20	74 x 74 x 119	Cl^-:46 K^+: 87	13,402

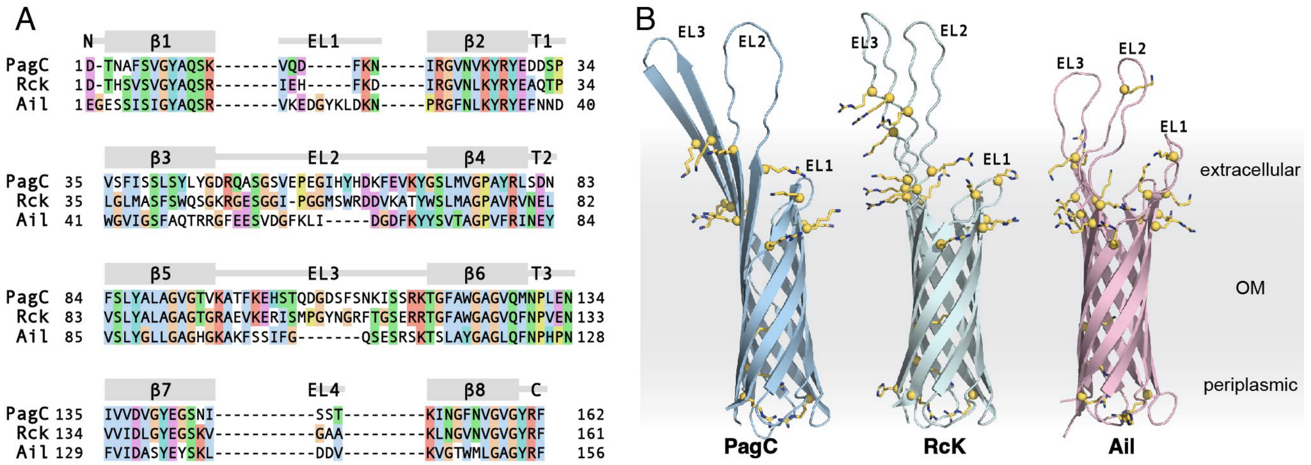

Figure S1. Sequence and structure of the PagC, RcK and Ail protein family.

(**A**) Structure-based sequence alignment of PagC, RcK and Ail. Alignments are rendered with ClustalX coloring using Jalview (3).

(B) Structural models of the proteins taken from AlphaFold (PagC, RcK) or determined experimentally (Ail), showing basic side chains (yellow stick with Carbon atom as yellow sphere).

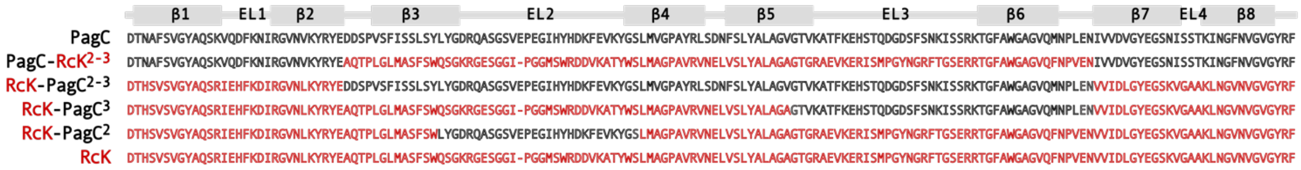

Figure S2. PagC-RcK chimeric mutants. The sequences of PagC (black) and Rck (red) chimeras were expressed in quadruple mutant *STm* (Δ*pagC*, Δ*rck*, Δ*ompX*, Δ*pgtE*) bacterial cells.

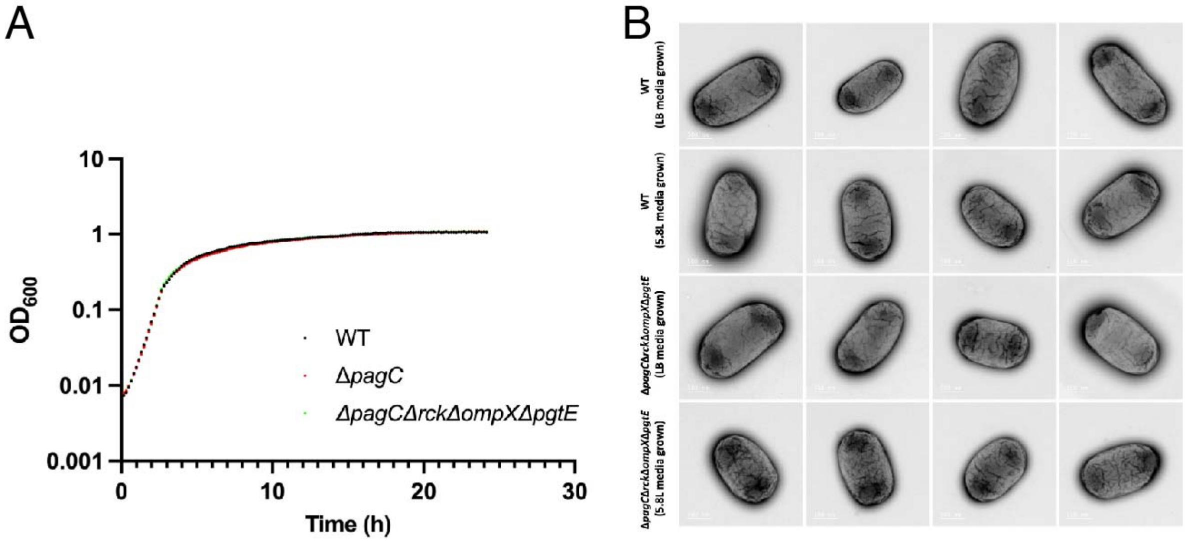

Figure S3. Growth comparison of Δ*pagC*, Δ*rck*, Δ*ompX*, Δ*pgtE* STm mutant strain with wildtype

(**A**) Growth curve of Wildtype (WT), Δ*pagC* and Δ*pagC*Δ*rck*Δ*ompX*Δ*pgtE* STm grown in LB media. Optical density at 600 nm (OD_{600}) was measured every 10 mins for 24 hours at 37℃, 180 rpm. Results represent three biological replicates.

(**B**) Four representative images of WT and Δ*pagC*Δ*rck*Δ*ompX*Δ*pgtE* STm captured by Transmission electron microscopy (TEM). The bacteria were grown in LB media or PhoPQ inducing 5.8L media, stained with 1% aqueous uranyl acetate and viewed under Tecnai 12 electron microscope.

A

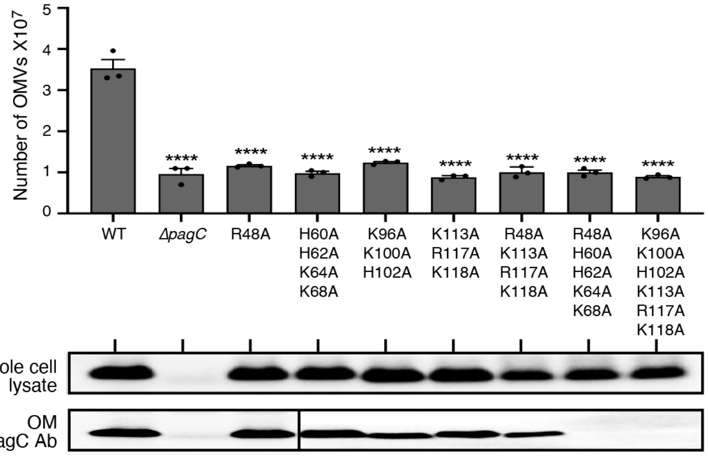

B

PagC	RcK	Ail
R48	R48	F54
H60	S59	F61
H62	R61	L63
K64	D63	D65
K68	T67	K69
K96	R95	K97
K100	K99	S101
H102	R101	I103
K113	T112	S107
R117	R116	S111
K118	R117	K112

Figure S4. PagC Ala substitution mutants.

(A) Number of OMVs produced by wildtype (WT) and PagC mutants, with Δ*pagC* serving as negative control. Mean values with standard error of the mean (s.e.m.) are shown (n = 3 biological replicates). Statistical significance was calculated using one-way ANOVA multiple-comparison test (****: $p < 0.0001$). Corresponding expression of PagC protein in whole cell lysate and bacterial OM fraction was detected by western blotting with anti-PagC antibody as the primary antibody.

(B) Correspondence of mutated PagC residues in the RcK and Ail sequences.

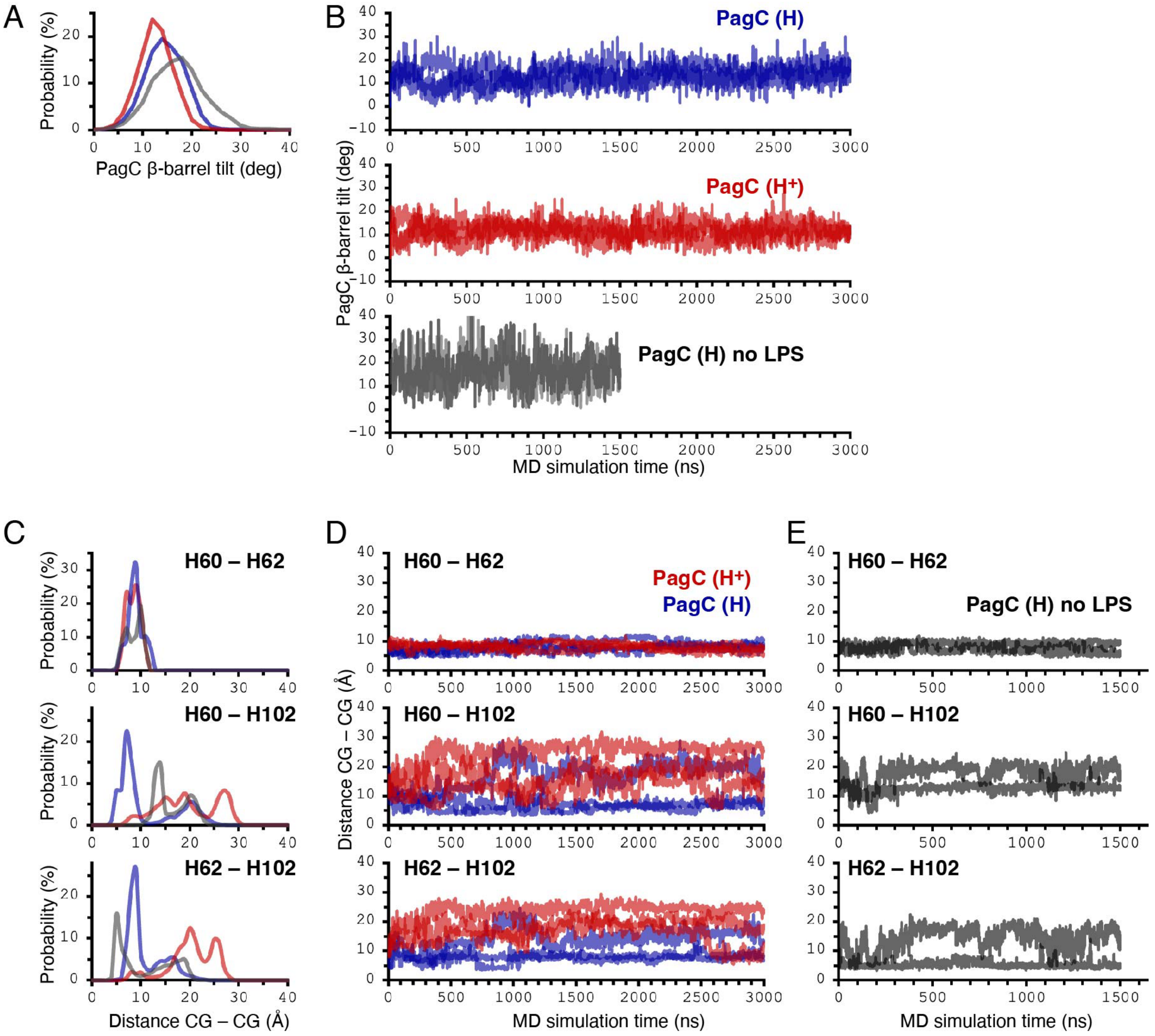

Figure S5. MD simulations of PagC.

(A, B) Probability distribution and time evolution of β-barrel tilt angle.

(C-E) Probability distribution and time evolution of His-His atomic distance. Data represent His-neutral (blue) and His-protonated (red) PagC in an STm OM, analyzed over the last 2 μs of three independent 3 μs MD simulations, or His-neutral PagC in a symmetric lipid bilayer (black), analyzed over the last 1 μs of two independent 1.5 μs MD simulations. Each time evolution trace is one independent MD simulation.

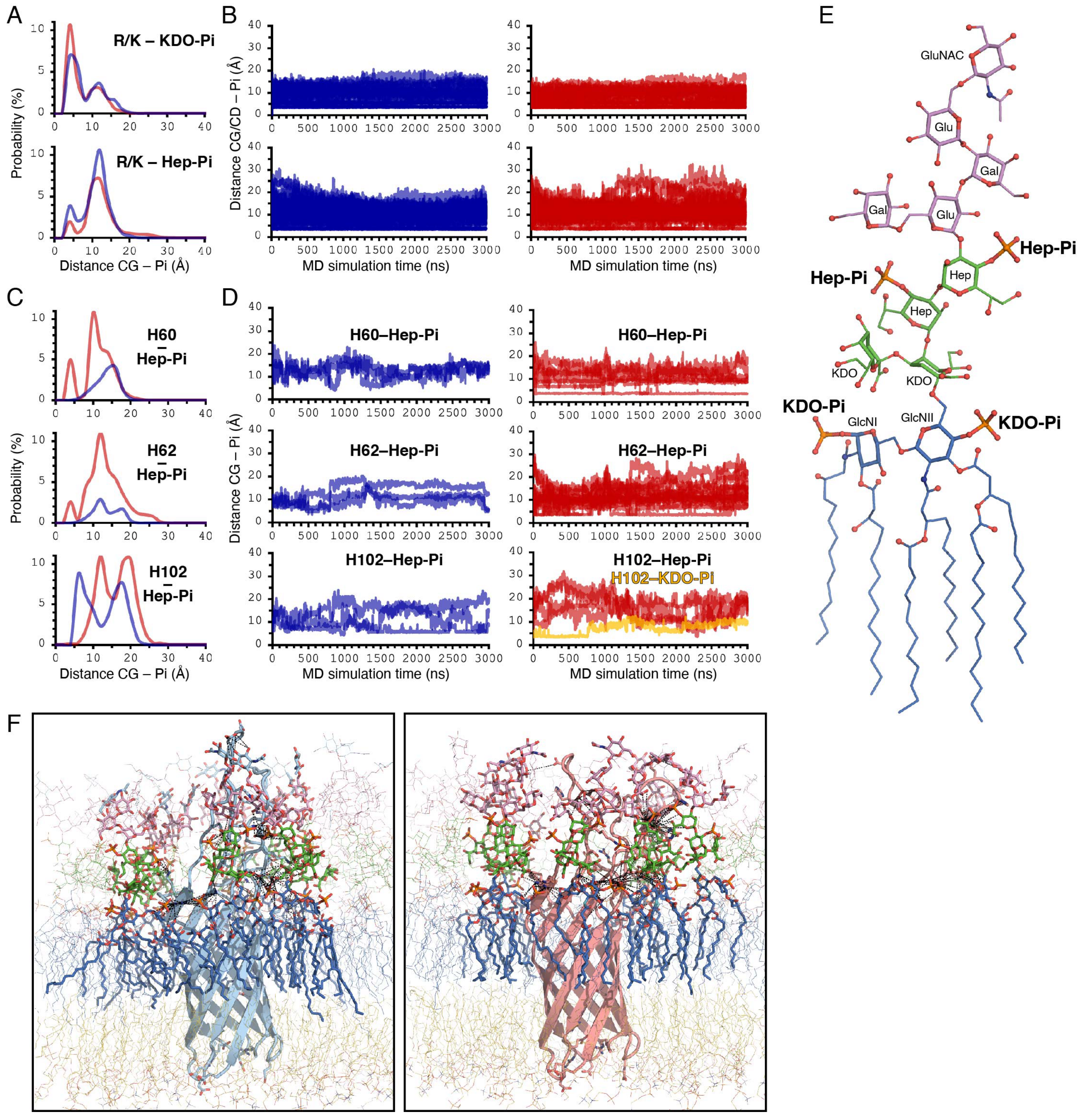

Figure S6. PagC–LPS Polar contacts established during MD simulation. Contacts between PagC Arg, Lys and His side chain atoms and LPS phosphate P atoms (Pi) were selected with a cutoff distance of 10 Å and a cutoff MD simulation lifetime longer than 100 ns. Data were obtained for His-neutral (blue) or His-protonated (red) PagC in an STm membrane. Data from the last 2 μs of each independent 3 μs MD simulation were analyzed.

(A, C) Probability density of PagC-LPS contacts per atomic distance. Probabilities are normalized to the total number of contacts formed by His-neutral and His-protonated PagC with LPS Pi, thus the area under each histogram reflects the number of LPS contacts made by each state of PagC. **(B, D)** Time evolution traces of the contact during the 3 μs MD simulation. **(E)** LPS molecular structure. Four Pi groups are marked, two from the KDO moiety and two from the inner core Hep moiety. **(F)** Representative structures taken at 3 μs of MD simulation for His-neutral (blue) or His-protonated (red) PagC. Associated LPS molecules are shown as sticks. Dashed lines between PagC and LPS denote polar contacts.

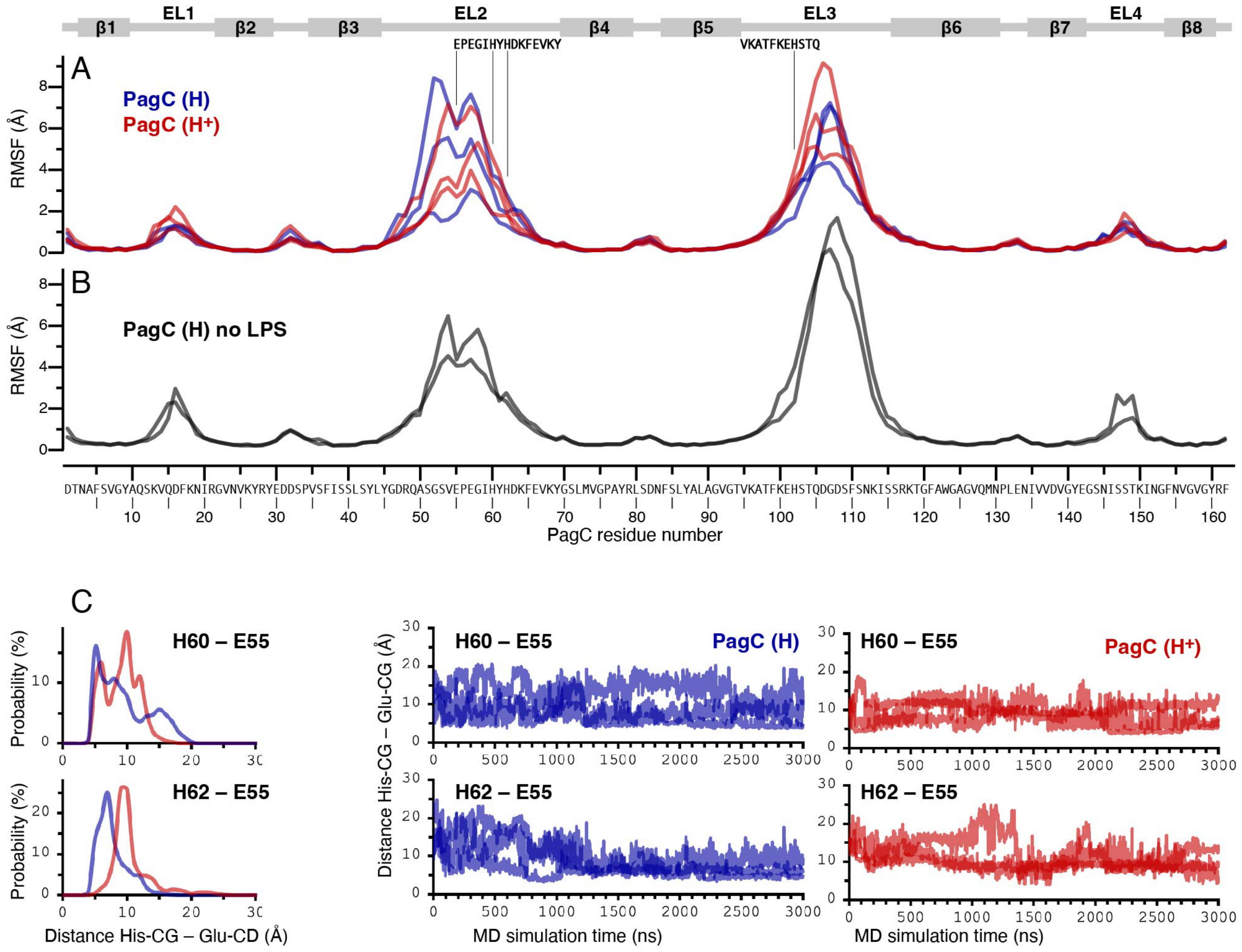

Figure S7. Time-averaged PagC residue contribution to RMSF. Time-averaged RMSF of PagC residues calculated for Carbon atoms. Protein secondary structure is shown above the data.

(A) Each trace is the time average over the last 2 μs for one independent 3 μs MD simulations of His-neutral (blue) or His-protonated (red) PagC.

(B) Each trace is the time average over the last 1 μs for one independent 1.5 μs MD simulation of His-neutral PagC in a symmetric lipid bilayer

(C) Probability distribution and time evolution of His-Glu55 atomic distances (between atoms His-CG – Glu-CD). Data represent His-neutral (blue) and His-protonated (red) PagC in an STm OM, analyzed over the last 2 μs of three independent 3 μs MD simulations. Each time evolution trace is one independent MD simulation.

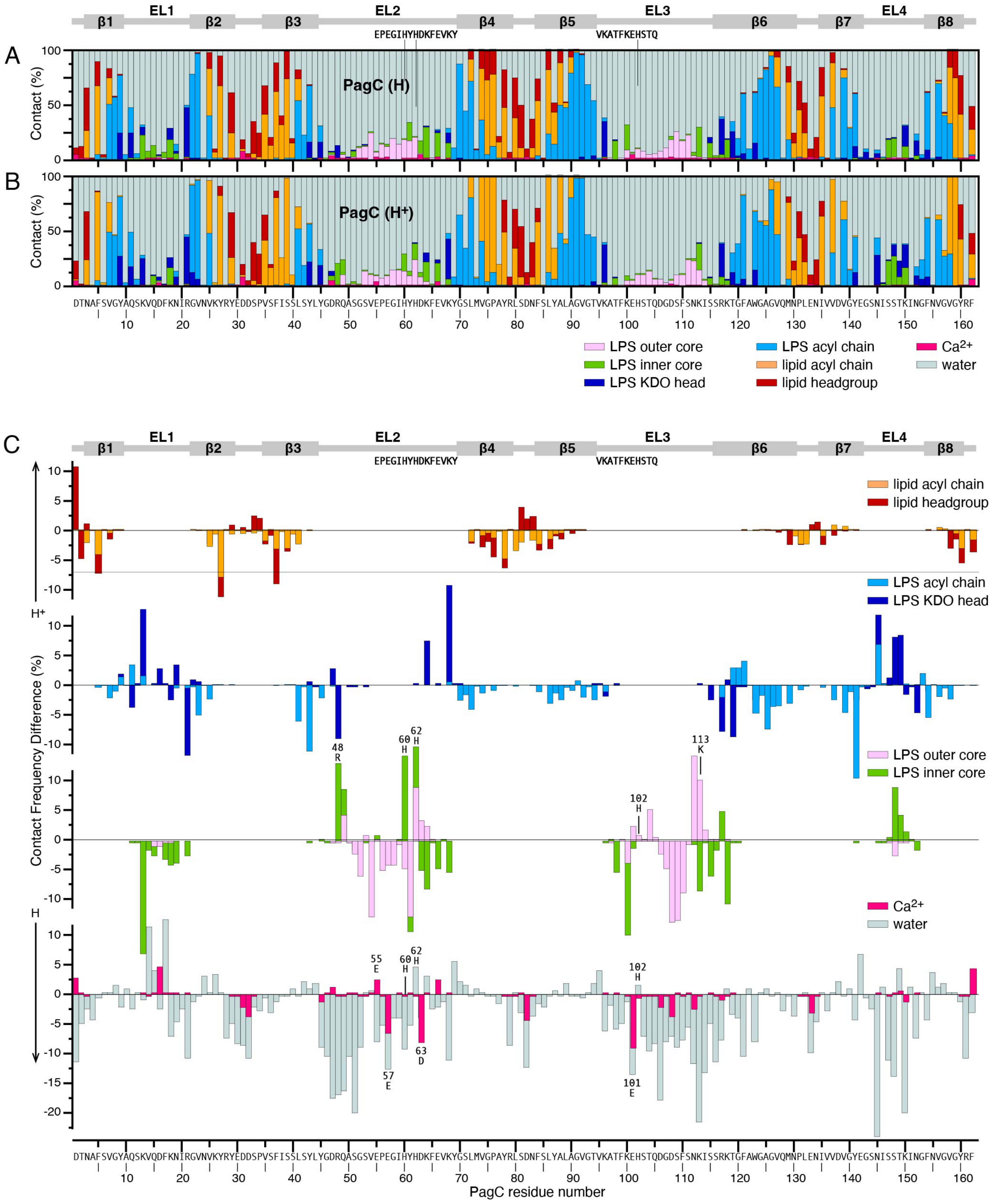

Figure S8. Time-averaged contacts of PagC residues with the Stm OM.

(A, B) Contact frequency for His-neutral (A) and His-protonated PagC (B). Each bar represents the frequency of 4 Å proximity to water, phospholipid moieties, LPS moieties, and Ca^{2+} ions. Interactions are averaged over the last 2 µs of 3 µs MD trajectories and three independent MD simulations. The protein secondary structure is depicted above the data.

(C) Difference in residue contacts of PagC residues induced by His protonation. Positive change reflects His protonation and negative change reflects His deprotonation.

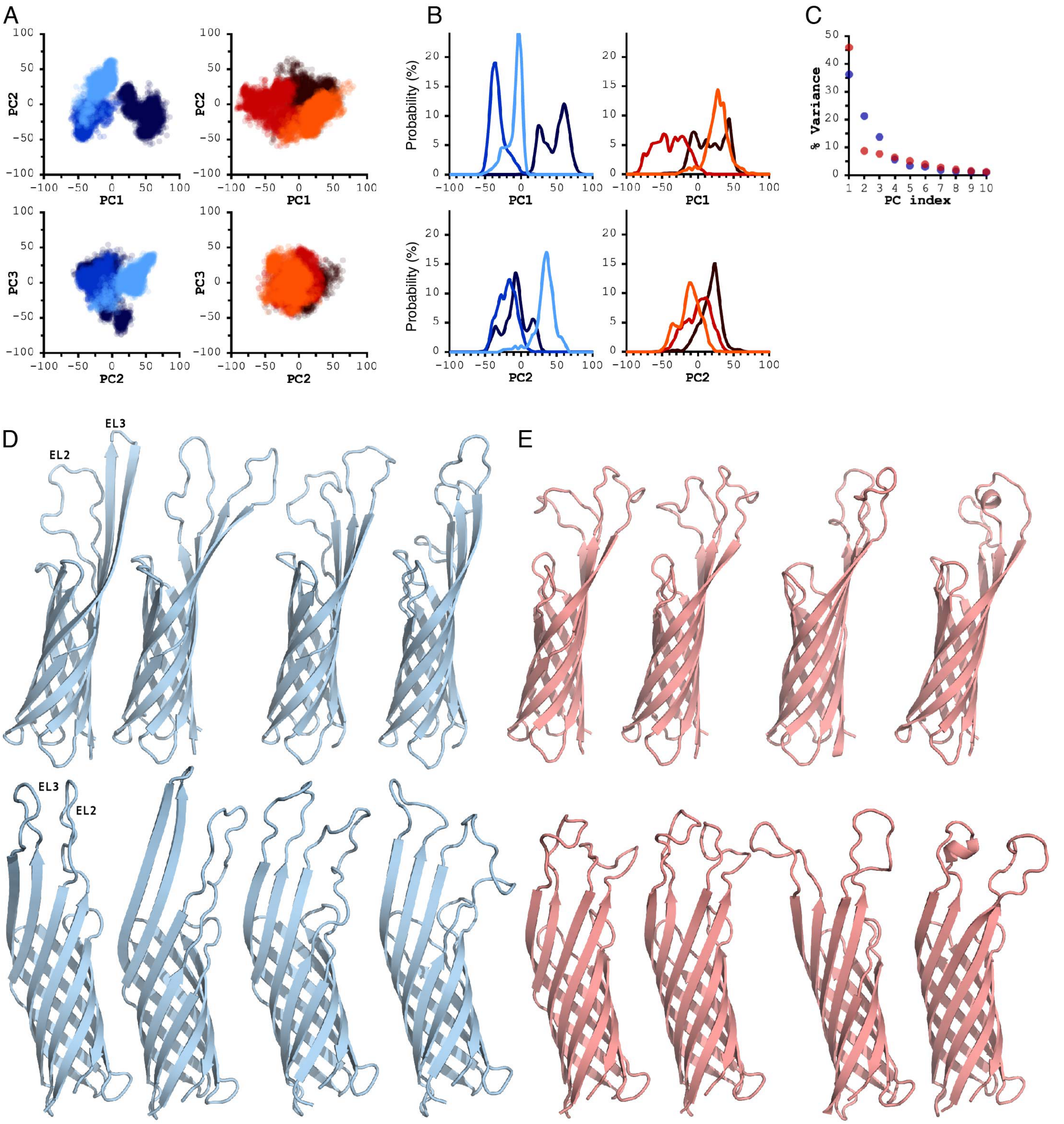

Figure S9. PC analysis of MD simulations. PC analysis was performed by concatenating three independent 3 μs MD simulations for either His-neutral (blue) or His-protonated (red) PagC. The data reflect variance of backbone heavy atom cartesian coordinates relative to the average of the concatenated structure.

(A, B) Conformer plots (A) and corresponding histograms (B) of PagC structures defined by the three principal components of the motion: PC1, PC2 and PC3. Each point represents a structure and point color indicates each of the three MD trajectories of His-neutral PagC (blue shades) or His-protonated PagC (Red shades).

(C) Plots of the % variance show that motion along PC1 is dominant.

(D, E) Representative structures taken from MD simulations of His-neutral (blue) or His-protonated (red) PagC at the specific values of the PC1 principal component of conformational exchange corresponding to populations in panel (B).

Supplementary References

1. R. Dehinwal *et al.*, Increased Production of Outer Membrane Vesicles by *Salmonella* Interferes with Complement-Mediated Innate Immune Attack. *mBio* **12**, e0086921 (2021).

2. K. Kawasaki, R. K. Ernst, S. I. Miller, Inhibition of *Salmonella enterica* serovar Typhimurium lipopolysaccharide deacylation by aminoarabinose membrane modification. *J Bacteriol* **187**, 2448-2457 (2005).

3. A. M. Waterhouse, J. B. Procter, D. M. Martin, M. Clamp, G. J. Barton, Jalview Version 2--a multiple sequence alignment editor and analysis workbench. *Bioinformatics* **25**, 1189-1191 (2009).
