# Supplementary material for: A pH-sensitive motif in an outer membrane protein activates bacterial membrane vesicle production
Source: Nat Commun. 2024 Aug 13;15:6958. doi: 10.1038/s41467-024-51364-z (PMC11322160; doi:10.1038/s41467-024-51364-z)
Supplement: Supplementary file 3 — Description of Additional Supplementary Files [file 41467_2024_51364_MOESM3_ESM.pdf]

Description of Additional Supplementary Files:

Supplementary Data 1: PagC and Rck protein models used for MD simulation studies
